# Brain Activity Response to Visual Cues for Gait Impairment in Parkinson’s Disease: An EEG Study

**DOI:** 10.1177/15459683211041317

**Published:** 2021-09-10

**Authors:** Samuel Stuart, Johanna Wagner, Scott Makeig, Martina Mancini

**Affiliations:** 1 Department of Sport, Exercise and Rehabilitation, 5995 Northumbria University, Newcastle upon Tyne, UK; 2 Northumbria Healthcare NHS foundation trust, North Tyneside, UK; 3 Department of Neurology, 6684 Oregon Health and Science University, Portland, OR, USA; 4 Swartz Center for Computational Neuroscience, Institute for Neural Computation (INC), 8784 University of California San Diego, San Diego, CA, USA

**Keywords:** electroencephalography, brain activity, walking, Parkinson’s disease, visual cues

## Abstract

*Background.* Gait impairments are common in Parkinson’s disease (PD) and increase falls risk. Visual cues can improve gait in PD, particularly freezing of gait (FOG), but mechanisms involved in visual cue response are unknown. This study aimed to examine brain activity in response to visual cues in people with PD who do (PD+FOG) and do not report FOG (PD-FOG) and explore relationships between attention, brain activity and gait. *Methods*. Mobile EEG measured brain activity during gait in 20 healthy older adults and 43 PD participants (n=22 PD+FOG, n=21 PD-FOG). Participants walked for 2-minutes with and without visual cues (transverse lines to step over). We report power spectral density (PSD) in Delta (1-4 Hz), Theta (4-7 Hz), Alpha (8-12 Hz), Beta (14-24 Hz) and Gamma (30-50 Hz) bands within clusters of similarly brain localized independent component sources. *Results*. PSDs within the parietal and occipital lobes were altered when walking with visual cues in PD, particularly in PD+FOG. Between group, differences suggested that parietal sources in PD, particularly with PD+FOG, had larger activity compared to healthy older adults when walking. Within group, visual cues altered brain activity in PD, particularly in PD+FOG, within visual processing brain regions. In PD participants, brain activity differences with cues correlated with gait improvements, and in PD+FOG those with worse attention required more visual attentional processing (reduced alpha PSD) in the occipital lobe. *Conclusions*. Visual cues improve gait and influence brain activity during walking in PD, particularly in PD+FOG. Findings may allow development of more effective therapeutics.

## Introduction

Gait impairments are a common and early feature of Parkinson’s disease (PD) and are a major cause of functional dependence, falls and mortality.^1^ PD gait impairments can be continuous (i.e. producing reduced stride length or gait speed) or intermittent (i.e. involving festination and/or freezing of gait (FOG)^2^). Although gait deficits in PD have largely been attributed to reduced dopamine within the nigro-striatal pathway, dopaminergic therapy has limited effect on gait.^3,4^ Recent studies have shown some efficacy of cholinergic therapies for gait improvement in PD,^5,6^ but evidence for pharmacological intervention for gait deficits remain limited. Therefore, non-pharmacological interventions such as cueing are commonly used to ameliorate gait impairments and reduce falls risk.^7^ Visual cues (e.g. transverse lines to step over) are often used to improve gait in PD,^8^ with theories suggesting visual^9^ and attentional^10^ mechanisms underpin the gait improvement. However, these theories of visual cue response are poorly understood and have received little investigation,^11,12^ despite the problem of variable response to cueing in clinical practice (i.e. some patients benefitting while others not).

The underlying neural mechanisms involved in gait impairment and response to visual cueing in PD are complex, with gait control not completely understood but thought to rely on contributions from multiple cortical and sub-cortical centres.^13^ The world is a visually complex place; it has been suggested that visual cues may help to focus attention on task-relevant visual processing (i.e. looking at the floor and stepping over lines, rather than attempting to process the entire visual environment),^14^ which could in itself help facilitate gait. It is therefore likely that the visual cueing response in PD^12^ is underpinned by both attentional and visual neural processing within relevant brain regions such as pre-frontal cortex and/or parietal or occipital regions. To date however, cortical brain activity underpinning visual cueing intervention response has not been examined in PD, which is likely due to the inability to image the brain while walking with traditional imaging techniques (e.g. functional magnetic resonance imaging).

Recent technological developments now allow monitoring of brain activity during real-time gait. Non-invasive, mobile electroencephalography (EEG) recording caps have recently been used to monitor primarily cortical activity during actual gait in those with PD,^15,16^ particularly those with PD and FOG,^17,18^ but electrophysiological differences to gait with and without visual cues are yet to be examined. One previous study has examined the effect of auditory cueing on reaction times in PD,^19^ but overall, few studies have examined EEG during gait. The few studies that have been conducted have demonstrated increased activity in theta and alpha bands in EEG sources localized to central (e.g. supplementary motor area; SMA) and frontal cortex, as well as stronger gamma band coherence over fronto-parietal-occipital pathways during FOG episodes compared to usual walking.^17,18^ However, our recent systematic review highlighted that previous EEG studies in PD have limited reports of brain activity outcomes to only a few channels (i.e. only involving electrodes placed at scalp sites Cz, Fz, P4 or Oz, referred to some other site).^20^ Brain source analysis is vital as the attribution of raw scalp channel recording features to individual brain areas is vague, at best, without it. That is, brain activity recorded by EEG at an individual channel may not represent activity beneath this channel location, with source localization (e.g. cluster analysis of channel components) being required to determine the origins of the recorded signal (e.g. some brain activity recorded by a scalp channel involving an electrode at Oz may originate from the frontal cortex).^21-23^

Previous studies have highlighted associations of cognitive function to selective gait outcomes in PD.^13^ For example, attention (involving PFC activation) has been related to control of pace-related gait characteristics such as step length and speed, whereas variability in visuo-spatial ability (involving parietal cortex) has been associated with gait variability and timing.^13^ Therefore, further work is needed to explore brain activity during walking recorded from multiple channels with group level source-resolved data analysis to provide a more exact understanding.

This study aimed to 1) examine brain activity in response to visual cues in healthy older adults and people with PD who do (PD+FOG) and do not report FOG (PD-FOG) and 2) explore relationships between brain activity and gait with visual cues, as well as cued brain activity and attention. We hypothesized that brain activity within cognitive (attentional) and visual processing brain regions would be altered with visual cues, particularly in PD+FOG. Additionally, we hypothesized that there would be reduced alpha and beta, as well as increased gamma band modulations within frontal and parietal regions when walking with visual cues compared to without, particularly in PD+FOG, as these EEG bandwidths are related to gait control in young adults^24-26^ and PD^16,27^ and to attention during cognitive or motor tasks.^28,29^ We also hypothesized that brain activity during gait with cues would relate both to cued gait and overall attentional capability.

## Methods

### Participants

A convenience sample of 20 healthy older adults and 43 participants with idiopathic PD (n=22 with self-reported FOG and n=21 without FOG) were recruited to this study. Participants with PD were recruited via neurologists at the Oregon Health and Science University (OHSU, Portland Oregon, USA) Movement Disorders Clinic. Self-reported FOG was based upon a question in the new Freezing of Gait Questionnaire after seeing the short clip related to the questionnaire.^30^ Subjects were categorized as ‘freezers’ if they have experienced such a feeling or episode over the past month. *Inclusion Criteria:* Clinical diagnosis of Parkinson’s by a movement disorder specialist according to UK brain bank criteria, Hoehn and Yahr stage II-III, aged ≥ 50 years, adequate vision and hearing and able to walk and stand unaided. *Exclusion Criteria:* Cognitive impairment (e.g. MoCA score <21), unstable medication for 1 month prior to study, psychiatric co-morbidity, acute lower back or lower/upper extremity pain, peripheral neuropathy, unable to comply with protocol and rheumatic and orthopaedic diseases affecting balance and gait.

### Protocol

Data were collected at the Balance Disorders Laboratory, OHSU, Portland, Oregon, USA, between July 2018 and November 2019. Each participant attended a 2-hour session at the laboratory in their usual ON medication state (within 60 minutes of taking anti-Parkinsonian medication).

### Clinical Assessment

Participants underwent a battery of demographic, clinical and cognitive assessments. Disease severity was measured using the Movement Disorder Society Unified Parkinson’s Disease Rating Scale Motor Subscale (MDS-UPDRS III),^31^ a 15-minute assessment of motor signs related to PD severity. Global cognition was assessed with the Montreal Cognitive Assessment (MoCA).^32^ Attention was measured with a computerized button pressing battery, involving simple (SRT) and choice reaction time (CRT), and digit vigilance (DV).^33^ Executive function was measured using Royall’s clock drawing (CLOX 1&2)^34^ and Trail-making Part B-A.^35^ Working memory and visuo-spatial ability were measured through seated forward digit span and judgement of line orientation (JLO)^36^ tasks, respectively. Basic visual functions of visual acuity and contrast sensitivity were assessed using standardized charts (logMar and logCS).

### Walking Assessment

Participants were first asked to stand as still as possible for 2 minutes while looking ahead. They were then asked to walk at a self-selected pace back and forth on a straight 9-m course (tape marked at either end) for 2-minutes under 2 different conditions: baseline (without cues) and with visual cueing (transverse black taped lines to step over). Visual cue distance was set to 20% above normal step length, in line with previous recommendations.^12^

Brain activity data were recorded at 2000 Hz during standing and walking using a 32-channel mobile EEG system (Mobita, TMSi), with electrodes placed according to the International 10–20 system. Locations of fiducial landmarks (nasion and left and right pre-auricular regions) were recorded (digitized) with a Polhemus Patriot 3D digitizer, which also provided 3D co-ordinates of the channel locations on the scalp. EEG channels included Fpz, Fp1, Fp2, Fz, F3, F4, F7, F8, FC1, FC2, FC5, FC6, AFz, T3, T4, Cz, C3, C4, TP7, CP3, CP4, CPz, TP8, T5, P3, Pz, P4, T6, PO7, PO8, O1, O2, each referenced to an average of all channels with a ground electrode attached to the forearm with a elasticated band.

Gait characteristics were measured using nine inertial measurement units (Opals Version 2, Mobility Lab Version 2, APDM Inc., Portland, OR, USA) strapped to both feet and shanks, to waist, to sternum, to both wrists and to the head.^37^

### EEG Data Analysis

EEG data were exported to a MATLAB (Mathworks, Natick, MA, USA) compatible format and then processed using the EEGLAB toolbox (14.1.2 Version, UC San Diego, Swartz Center for Computational Neuroscience (SCCN), La Jolla, CA, USA).^22^ EEG data from separate 2-minute standing, walking and walking with visual cues were concatenated within MATLAB, providing a 6-minute trial to process (this allowed multiple datasets in the same session to be used to identify independent component (IC) processes (weights and locations) across conditions Walk and Cue). The continuous EEG data were then down-sampled from 2000 Hz to 500 Hz and band-pass filtered in the 1–200 Hz range using the FIR filter (order = 1650) function. The *cleanLineNoise* function (within the PREP pipeline function) then was used to reduce EEG signal noise, with tau set to 100, pad set to 2, line frequencies specified (60, 120, 180 and 240 Hz), a significance cut-off level for removing spectral peaks of *P*=.01 and 2-Hz scan/taper frequency bandwidth (within a 4-second window size using a 1-second sliding window length).^38^ Since the PREP pipeline function does not prune EEG data within noisy (‘bad’) recording epochs, visual inspection was also conducted to remove ‘bad’ data segments.^39^ The *clean_rawdata* function was then used to remove bad channels (with artefact classed as a channel remaining flat for more than 5 seconds, high frequency noise over 4 SDs or minimum correlation between neighbouring channels below .85)^40^ and to correct the continuous data using Artefact Subspace Reconstruction (ASR) (with max acceptable 0.5-second window SD of 20).^41^ The channel data were then average referenced.

### Independent Component Decomposition and Dipole Source Analysis

Decomposition of the data by independent component analysis (ICA) was performed within EEGLAB on individual subject datasets concatenating data from all conditions (concatenated Stand, Walk, Visual Cue). This separated the EEG signals into maximally independent component (IC) processes. The *dipfit* function within EEGLAB was then used to derive the single equivalent dipole model that best explained scalp topography of each IC using a boundary element electrical head model based on the Montreal Neurological Institute (MNI) template (http://www.mni.mcgill.ca).^42^ ICs with model residual scalp map variance (RV) above 15%, or whose model equivalent dipole was located outside of the MNI head model space, or had a scalp topography or power spectrum that indicated an artefactual source were excluded at the individual participant level. Component exclusions based on scalp topography and power spectrum were made using visual inspection and the *ICLabel* function. ICs were included in further analysis if classified by ICLabel as >70% likelihood of being brain activity rather than artefact, having RV <15%, and whose model equivalent dipole was located within MNI brain volume).^43^

A joint measure IC pair distance measure was built using relative weights: (1–200 Hz) power spectral difference, 6; scalp map difference, 2, and dipole location difference, 12. The resulting IC joint-measure vectors were reduced to 10 dimensions by principal component analysis (PCA).

### Component Clustering Across Subjects

A robust K-means method was employed for IC clustering, which used Euclidean distance between ICs in the joint measure space to find, first, IC cluster centroids^44-46^ and then to cluster ICs near to the IC cluster centroids. Outlier components more than 3 standard deviations from any IC cluster centroid were assigned to an outlier IC cluster and excluded from further analyses.^47^ Further visual inspection of the spectra of the ICs within each cluster allowed removal of suspected artefact sources (e.g. accounting for scalp muscle activity) to the outlier IC cluster. On average, 25.3% (n=94) of the ICs were classified as outliers, a percentage similar to those reported in previous EEG gait studies using ICA decomposition.^48^

The optimum number of final clusters was determined by first starting with a small number of clusters (the smallest number in which activation properties were not merged across clusters) and then increasing the number of clusters by 1.^48^ Following the addition of an extra cluster, if IC dipole groups were separated into two but other IC properties (and the differences between the groups and conditions) remained approximately the same, then the maximum number of clusters was deemed to have been exceeded. In this way, six distinct IC clusters were identified, which is consistent with other mobile EEG clustering techniques where the number of ICs (n=371) is divided by the total number of subjects (n=63) (i.e. 371/63 = 6).^49^ As there is no official consensus on IC cluster formation, clusters were retained for further analysis if they contained approximately half (rounded to the nearest ten) of the participants in each cluster, in line with previous mobile EEG research.^49^

The primary outcome measures were power spectral density (PSD; log-transformed) in 5 bands: Delta (1–4 Hz), Theta (4–7 Hz), Alpha (8–12 Hz), Beta (14–24 Hz) and Gamma (30–50 Hz).

### Statistical Analysis

Demographic, gait and correlational data were analysed using SPSS (IBM Inc, USA) and assessed for normality using Kolmogorov–Smirnov tests with data meeting the criteria for parametric analysis. One-way analysis of variance (ANOVA) was used to compare continuous demographic variables across all groups (HC, PD-FOG, PD+FOG), and t-tests were used to compare individual group comparisons (HC vs PD-FOG, HC vs PD+FOG, PD-FOG vs PD+FOG), and chi-square analysis for ordinal data. The significance level was a priori set at *P*<.05.

*EEG Data:* EEG statistical analysis involved assessment of differences in PSDs between groups (HC, PD-FOG, PD+FOG) and conditions (walk, cue) with permutation statistics (2000 permutations) with a 95% confidence interval (*P*<.05), within the MATLAB EEGLAB toolbox. Type I error was controlled by correcting conservatively for false discovery rate (FDR). For overall effects, analysis involved a permutation based repeated measures (3×2) ANOVA design (*Group*: HC, PD-FOG, PD+FOG, *Condition*: Walk, Visual Cue). *Within Group Effects:* Post-hoc paired permutation t-tests were used to investigate differences between conditions (Walk, Visual Cue) within each group (HC, PD-FOG, PD+FOG), with FDR adjustment for multiple comparison. *Between Group Effects:* Post-hoc permutation based one-way repeated measures ANOVAs (1×3) were used to assess overall difference between groups (HC, PD-FOG, PD+FOG) separately within each condition (Walk, Visual Cue), with FDR adjustment for multiple comparison. Further separate paired permutation t-tests examined individual group differences (HC vs PD-FOG, HC vs PD+FOG, PD-FOG vs PD+FOG), with FDR adjustment for multiple comparison.

*Gait Data:* To examine changes in gait with visual cues from baseline, we used separate repeated measures (1×3) ANOVAs for each gait characteristic within SPSS, which compared the effect of cueing (Walk, Visual Cue), with group (HC, PD-FOG, PD+FOG) as a between subject factor. Post-hoc Tukey HSD tests were performed to examine differences between specific groups (HC vs PD-FOG, HC vs PD+FOG, PD-FOG vs PD+FOG).

**Figure 1.**
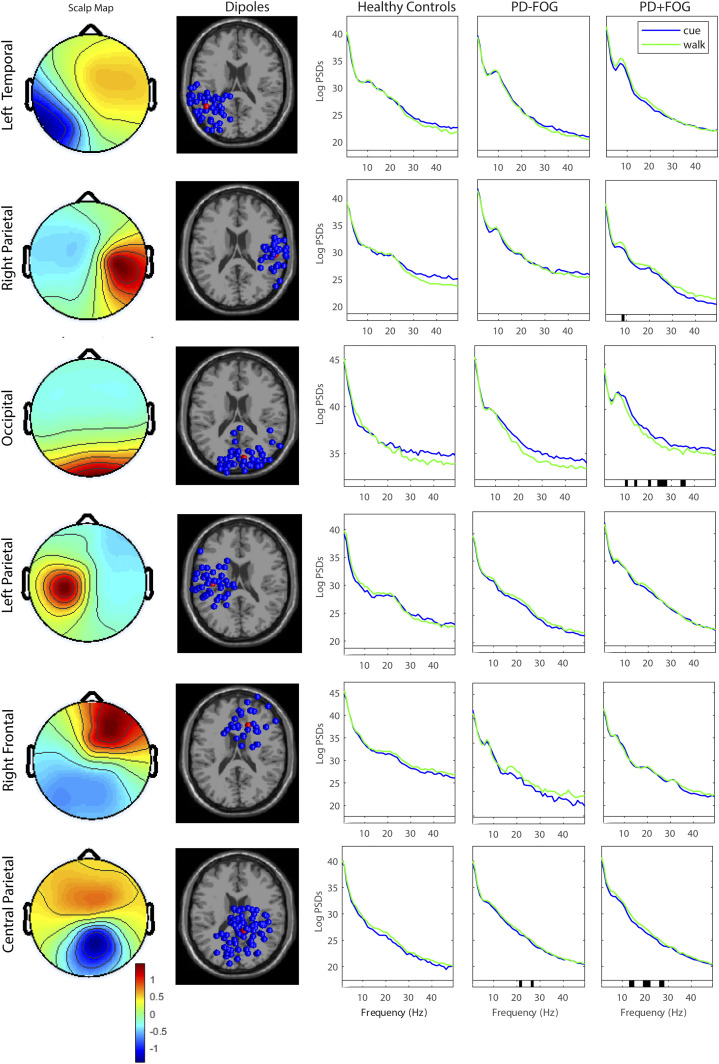
Power Spectral Densities in Brain Cluster Locations in people with PD (PD-FOG and PD+FOG) and healthy controls when walking with and without visual cues [Scalp maps, dipole cluster locations and Log PSDs, Significant (*P* < .05) differences in PSDs denoted by black bar on x axis].

*Brain Activity and Gait Relationships:* To explore relationships between cued brain activity (average PSDs of significantly different bandwidths with cues) and gait (gait speed, stride length, foot strike angle and double support time) with visual cues, as well as cued brain activity and attention (SRT, CRT and DV) in PD, we used separate Spearman’s rho correlations within SPSS due to the exploratory nature of the analysis and the reduced number of IC clusters analysed. Only PSDs in brain regions that were significantly different with visual cueing were entered into correlational analysis (Figure 1).

## Results

### Participants

Table 1 shows that the three groups (HC, PD-FOG, PD+FOG) were well matched for age (*P*=.535), sex (*P*=.350), depression (GDS-15; *P*=.128), global cognition (MoCA, *P*=.426) and vision (acuity; *P*=.657, contrast sensitivity; *P*=.869). PD+FOG had significantly worse disease severity (UPDRS III; *P*=.001), longer disease duration (*P*=.021) and greater levodopa medication dosage than PD-FOG (*P*=.009). Additionally, PD+FOG had significantly worse attention than HC and PD-FOG, as measured via choice reaction time (*P*=.0.31) and digit vigilance (*P*=.037).

**Table 1.** Participant Demographic and Clinical Outcomes.

	HC (n=20)	PD-FOG (n=21)	PD+FOG (n=22)				
	Mean (SD)	Mean (SD)	Mean (SD)	*P* ^a^	*P* ^b^	*P* ^c^	*P* ^d^
Age	69.40 (8.05)	69.33 (4.97)	68.09 (7.69)	.535	.836	.490	.651
Sex	M 11/F 9	M 11/F 10	M 15/F 7	.350	1.00	.358	.358
Height (m)	1.70 (.09)	1.69 (.11)	1.74 (.09)	.250	1.00	.090	.109
Weight (kg)	74.39 (13.84)	76.10 (19.80)	78.78 (16.86)	.150	.515	.011*	.105
GDS-15	3.15 (4.11)	4.95 (5.10)	7.18 (4.28)	.128	.219	.003*	.130
Education	17.65 (2.54)	17.14 (2.76)	16.55 (2.50)	.461	.521	.147	.541
Falls past 12months (n)	.50 (1.10)	.33 (.58)	6.05 (11.50)	.028*	.596	.034*	.030*
Handedness	R 18/L 3	R 21/L 2	R 22/L 0	.202	.232	.664	.488
MoCA	27.10 (2.15)	27.62 (2.22)	27.00 (2.78)	.426	.480	.850	.415
CLOX1	13.25 (1.41)	12.86 (1.68)	12.68 (1.64)	.731	.422	.235	.623
CLOX2	13.45 (1.47)	13.95 (1.24)	13.86 (.83)	.784	.246	.277	.798
JLO	26.40 (3.56)	24.81 (4.68)	22.96 (8.24)	.372	.227	.085	.398
Digit span	5.95 (.95)	6.29 (1.23)	5.91 (1.11)	.297	.479	.774	.299
SRT (ms)	338.96 (43.02)	351.12 (66.18)	395.29 (85.74)	.067	.479	.010*	.063
CRT (ms)	508.18 (66.71)	519.59 (97.50)	616.65 (174.80)	.031*	.566	.009*	.032*
DV (ms)	466.72 (40.46)	490.72 (66.86)	543.09 (90.28)	.037*	.180	.001*	.025*
Visual acuity (LogMar)	.04 (.13)	.06 (.18)	.08 (.11)	.657	.636	.242	.623
Contrast sensitivity (LogCS)	1.66 (.13)	1.57 (.20)	1.58 (.13)	.869	.115	.057	.929
Disease duration (years)	—	5.50 (2.95)	9.48 (7.00)	—	—	—	.021*
H&Y stage	—	I (0)/II (21)/III (1)	I (0)/II (19)/III (4)	—	—	—	.170
LEDD (mg)	—	628.64 (372.81)	945.42 (385.83)	—	—	—	.009*
MDS-UPDRS III	—	25.38 (12.34)	38.86 (12.95)	—	—	—	.001*
FOGQ	—	.00 (.00)	14.77 (6.96)	—	—	—	<.001*

^a^All Participants ANOVA.

^b^HC vs PD-FOG *t*-test.

^c^HC vs PD+FOG *t*-test.

^d^PD-FOG vs PD+FOG *t*-test.

CLOX1&2, Royall’s clock drawing task; CRT, choice reaction time; DV, digit vigilance; FOGQ, Freezing of Gait Questionnaire; GDS-15, Geriatric Depression Scale (short form); H&Y, Hoehn and Yahr; JLO, judgement of line orientation; LEDD, levodopa equivalent daily dosage; MDS-UPDRS III, Movement Disorder Society Unified Parkinson’s Disease Rating Scale (motor sub-section); MoCA, Montreal Cognitive Assessment; SRT, simple reaction time. *P<.05.

### ICA Clustering

Overall, 371 ICs (6.0**±**2.6 per subject) were retained across the three groups (HC, PD-FOG, PD+FOG). IC clustering analysis produced 6 active clusters (plus an outlier cluster) that included 277 ICs (4.5**±**2.5 per subject), not including the outlier cluster. Table 2 shows the cluster locations, number of subjects per cluster, corresponding Brodmann area (BA) and MNI co-ordinates for each cluster centroid. The cluster centroids were located within the left temporal lobe, right, left and central parietal lobe, occipital lobe and frontal lobe, respectively.

**Table 2.** Localizations of Cluster Centroids are Shown in Talairach Co-ordinates Along with the Corresponding Nearest Grey Matter Locations and Brodmann Areas.

			Centroid Co-ordinates				
	No. of Subjects	No. of ICs	X	Y	Z	BA	Grey Matter Location
*Cluster 1*	Total = 33	47	−48	−52	7	22	Middle temporal gyrus
Left temporal	HC = 9	—	—	—	—	—	Superior temporal gyrus
	PD-FOG = 11	—	—	—	—	—	—
	PD+FOG = 13	—	—	—	—	—	—
*Cluster 2*	Total = 30	33	55	−22	33	6	Post-central gyrus
Right parietal	HC = 10	—	—	—	—	—	Pre-central gyrus
	PD-FOG = 10	—	—	—	—	—	—
	PD+FOG = 10	—	—	—	—	—	—
*Cluster 3*	Total = 37	44	11	−82	−21	18	Lingual gyrus
Occipital	HC = 10	—	—	—	—	—	Fusiform gyrus
	PD-FOG = 16	—	—	—	—	—	—
	PD+FOG = 11	—	—	—	—	—	—
*Cluster 4*	Total = 37	46	−40	−18	48	40	Post-central gyrus
Left parietal	HC = 9	—	—	—	—	—	Inferior parietal lobule
	PD-FOG = 16	—	—	—	—	—	—
	PD+FOG = 12	—	—	—	—	—	—
*Cluster 5*	Total = 25	30	16	26	31	32	Medial frontal gyrus
Right frontal	HC = 10	—	—	—	—	—	Cingulate gyrus
	PD-FOG = 9	—	—	—	—	—	—
	PD+FOG = 6	—	—	—	—	—	—
*Cluster 6*	Total = 48	77	11	−40	63	4	Post-central gyrus
Central parietal	HC = 11	—	—	—	—	—	Paracentral lobule
	PD-FOG = 17	—	—	—	—	—	—
	PD+FOG = 20	—	—	—	—	—	—

BA, Broadman areas; IC, independent component.

### In PD, Walking With Visual Cues Changes Spectral Power in Parietal and Occipital Sources

*Within group effects:* Figure 1 displays the scalp maps, dipoles and PSDs of the six clusters during walking with and without cues within the three groups: HC, PD-FOG and PD+FOG. For healthy controls, walking with versus without visual cues did not change any PSDs within any cluster source (Figure 1, middle column). For PD+FOG, sources in the right parietal lobe cluster produced lower alpha band power (T=−3.42, *P*=.037) when walking with visual cues compared to walking without cues (Figure 1, second row, right column).

When walking with visual cues, there were also significant reductions in beta band power in central parietal lobe sources of PD+FOG participants (Figure 1, bottom row, right column) (T=−2.78, *P*=.045) and in the upper beta band for both PD groups (PD-FOG, T=−4.14, *P*=.01; PD+FOG, T=−3.59, *P*=.025). Additionally, in PD+FOG alpha (T=3.57, *P*=.041), beta (T=3.18, *P*=.044) and gamma (T=3.72, *P*=.025) power in occipital lobe cluster sources was lower when walking with visual cues than without cues.

**Figure 2.**
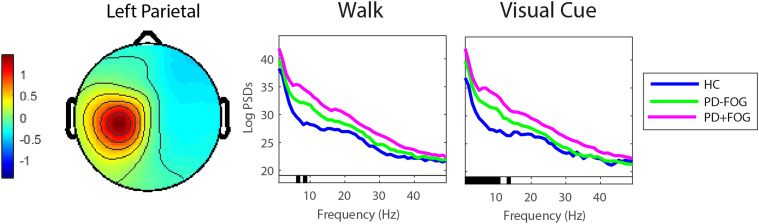
Power spectral density of the left parietal cortex when walking without and with visual cues in people with PD (PD-FOG, PD+FOG) and health controls [significant (*P*<.05) differences in PSDs denoted by black bar on x axis].

*Between group effects:* Figure 2 shows group comparisons between left parietal sources. Overall, the left parietal source for healthy controls produced significantly less theta (F=−5.19, *P*=.025) and alpha band power (F=−4.42, *P*=.037) during walking compared to PD groups (both PD-FOG and PD+FOG). While walking with visual cues, healthy control sources in the left parietal cluster produced less power in the delta (F=5.18, *P*=.034), theta (F=5.20, *P*=.034) and alpha (F=4.68, *P*=.038) bands than sources of PD participants (both PD-FOG and PD+FOG). There were no significant pairwise (t-test) differences between PD-FOG and PD+FOG, or group (HC, PD-FOG, PD+FOG) by condition (cue/no cue) interactions for any of the IC clusters.

### Gait Improves with Visual Cues and Relates to Brain Activity in PD

As expected, gait characteristics were significantly improved with the application of visual cues in all groups (HC, PD-FOG, PD+FOG) (Table 3). Exploratory correlational analysis showed that brain source power differences in PD participants walking with visual cues were moderately correlated with selective measures of gait performance. Specifically, in PD-FOG foot strike angle was positively correlated with beta band power in central parietal sources (rho=.669, *P*=.003, Table 4), and in PD+FOG gamma band power was positively correlated with gait speed (rho=.608, *P*=.036, Table 4) and with longer stride length (rho=.58, *P*=.050). Additionally, in PD+FOG occipital alpha band source power was negatively correlated with attentional reaction time (rho=−611, *P*=.016, Table 4).

**Table 3.** Gait Characteristics With and Without Visual Cues.

							Repeated Measures ANOVA			Tukey HSD – *Post hoc*		
	HC		PD-FOG		PD+FOG		Cue (Walk, Visual Cue)	Group (HC, PD-FOG, PD+FOG)	Cue x Group	HC vs PD-FOG	HC vs PD+FOG	PD-FOG vs PD+FOG
	Mean (SD)		Mean (SD)		Mean (SD)							
	Walk	Visual Cue	Walk	Visual Cue	Walk	Visual Cue	*F (p)*	*F (p)*	*F (p)*	*p*	*p*	*P*
Gait speed (m/s)	1.13 (.15)	1.21 (.16)	1.07 (.14)	1.10 (.17)	.89 (.16)	.93 (.19)	33.82 (<.001*)	15.01 (<.001*)	2.54 (.088)	.202	<.001*	.001*
Stride length (m)	1.19 (.09)	1.35 (.11)	1.12 (.14)	1.29 (.14)	.98 (.15)	1.14 (.17)	349.17 (<.001*)	15.04 (<.001*)	.20 (.818)	.237	<.001*	.001*
Foot strike angle (°)	18.38 (3.08)	22.68 (2.85)	15.23 (4.24)	19.08 (4.75)	9.82 (5.32)	15.34 (1.25)	144.24 (<.001*)	20.65 (<.001*)	1.82 (.170)	.020*	<.001*	<.001*
Double support time (ms)	20.66 (3.43)	18.94 (3.13)	20.48 (2.41)	19.05 (2.73)	22.36 (4.50)	21.64 (4.17)	31.84 (<.001*)	3.17 (.049*)	1.72 (.187)	.999	.114	.101

*P<.05. ANOVA, analysis of variance; HC, healthy control; PD-FOG, Parkinson’s disease without freezing of gait; PD+FOG, Parkinson’s disease with freezing of gait; SD, standard deviation.

**Table 4.** Relationship Between Brain Activity, Attention and Gait with Visual Cues in PD.

	PD-FOG		PD+FOG			
	Cluster 6	Cluster 2	Cluster 3			Cluster 6
	Central Parietal	Right Parietal	Occipital			Central Parietal
	Beta	Alpha	Alpha	Beta	Gamma	Beta
Gait	—	—	—	—	—	—
Gait speed	.444 (.074)	−.176 (.627)	.357 (.255)	.490 (.106)	.608 (.036)*	−.060 (.801)
Stride length	.382 (.130)	−.128 (.725)	.351 (.263)	.467 (.126)	.575 (.050)*	−.229 (.330)
Foot strike angle	.669 (.003)*	.212 (.556)	−.028 (.931)	−.014 (.966)	.042 (.897)	.038 (.875)
Double support time	−.395 (.117)	.491 (.150)	−.329 (.297)	−.490 (.106)	−.531 (.075)	.182 (.443)
Attention						
SRT	−.466 (.060)	.297 (.405)	−.450 (.092)	.132 (.639)	.214 (.443)	−.294 (.252)
CRT	−.179 (.492)	.503 (.138)	−.393 (.147)	.111 (.694)	.039 (.889)	−.147 (.573)
DV	−.091 (.729)	.321 (.365)	−.611 (.016)*	−.214 (.443)	−.057 (.840)	−.216 (.406)

Spearman’s rho (p).

*P<.05. CRT, choice reaction time; DV, digit vigilance; PD-FOG, Parkinson’s disease without freezing of gait; PD+FOG, Parkinson’s disease with freezing of gait; SRT, simple reaction time.

## Discussion

To the best of our knowledge, this study is the first to examine brain activity when walking with and without visual cues in healthy older adults and in PD adults with and without freezing of gait (FOG). Across all groups, brain source activities during walking with and without visual cues were identified as originating in the frontal, temporal, parietal and occipital lobes. Within-group findings suggest that in PD participants, and particularly in PD participants showing freezing of gait (FOG), use of visual cueing affected brain activities within spatial and visual processing brain regions. Specifically, parietal and occipital brain sources exhibited significant differences in alpha, beta and gamma band power. These EEG power differences may be related, directly or indirectly, to the improved gait of PD participants while using visual cues as the power differences correlated significantly to more than one gait feature for both PD groups. Between-group findings suggest that when walking with visual cues, parietal brain sources of people with PD, particularly those with freezing of gait, produce more theta, delta and alpha brain activity than healthy older adults. This may indicate greater reliance on or application of resources required for visual processing in PD and particularly in PD+FOG. Additionally, for the PD+FOG group, poorer attention (i.e. digit vigilance) was associated with less occipital source alpha activity while walking with visual cues, suggesting that persons with PD and freezing of gait, and particularly those with slower manual response times, may devote more visual attention when walking with visual cues than PD subjects without freezing of gait.

### Brain Activity When Walking: Response to Visual Cues

Visual cues altered brain activity during gait in those with PD, but not healthy older adult controls. Overall, findings suggest that people with PD have higher PSDs (within selective bands) than controls within sensory regions (parietal and occipital lobes) when walking with visual cues, which would suggest that the cues elicit heightened sensory feedback for integration into walking. Visual cues may therefore reduce the burden of walking in PD, particularly PD+FOG, by influencing sensory processing of task relevant information (i.e. lines on the floor to step over),^12^ which helps to ameliorate walking deficits. Interestingly, in opposition to the theories of frontal attentional or executive brain regions being principally involved in visual cue response, the cues did not significantly influence total frontal brain region independent source activity, which may reflect the intact cognition seen within the groups. However, better time-resolved time–frequency measures including source network coherence might better resolve group EEG source differences during walking.^25,50^

In relation to sensory region processing, there were significant differences in brain activity of PD participants in the parietal and occipital lobe sources between walking with and without visual cues, particularly for PD+FOG, which may reflect regional changes in visual, cognitive and motor processing. Specifically, in parietal lobe sources of PD+FOG participants produced significantly less alpha power when they walked with rather than without visual cues; this may indicate an increase in externally directed attention towards the cues.^51^ In a previous auditory cueing study in which healthy young adults showed a similar source-level alpha reduction in the parietal cortex during walking, the authors suggested this might relate to their visual attention being applied to the distance or time to the next cue stimulus.^25^ In another study with healthy young adults, increased visual input also reduced source-level parietal alpha power during walking, particularly when visual input was related to participant movement.^50^ Additionally, other studies have reported increased parietal alpha power in PD subjects during FOG episodes.^52^ In our data, alpha power in occipital lobe sources in our PD+FOG participants was also reduced during visually cued walking, again compatible with greater attention to visual cues in this group^51^ during increased visual demand.^53^

Interestingly, previous studies of balance motor tasks in older adults have shown that gamma and beta band activity increases in scalp-level parietal and occipital regions may facilitate sensorimotor integration of external sensory feedback.^53^ We also observed more gamma power in occipital lobe sources of PD+FOG participants during visual cue walking, which may reflect visuomotor processing of the cues in this group^54^ linked to other cognitive processes such as visual short-term memory and selective attention.^55,56^ This gamma band difference in PD+FOG is similar to those observed in healthy adults during walking with tactile stimulus^57^ and in PD subjects in response to obstacle crossing (in the Oz scalp channel).^16^

Higher occipital beta band power in our PD+FOG participants during cued walking is likely related to their need for increased visual attentional processing^58^ and use of the additional visual information^59^ to complete the task. The smaller parietal (source localized) beta band power in PD participants walking with visual cues (with or without FOG symptoms) is similar to results of a recent mobile EEG study of obstacle crossing in PD that also reported reduced beta power in a CPz scalp channel over parietal regions during obstacle crossing.^16^ In healthy young adults, a reduction in beta power has been reported during balance beam walking,^26^ which other studies suggest may relate to increased sensory processing due to changes to visual input.^60^ Beta power reduction during cued walking may also indicate stronger motor preparation to perform the task,^61^ for example, while using the additional visual information (the lines on the floor) to plan the walk more effectively.^62^

Previous mobile EEG studies in PD have been limited to examination of only a limited number of subjects and EEG channels for analysis (typically, 1–4 channels)^20^; this has limited group comparison and interpretation. In the current study, we were able to examine brain activity with higher spatial resolution in a larger cohort, allowing a three-group (HC, PD+FOG, PD-FOG) comparison. We found that people with PD, particularly PD+FOG, had significantly higher left parietal lobe activity levels in several EEG bands (theta, alpha and delta) compared to healthy older adults when walking with rather than without visual cues. Previous imaging studies using magnetic resonance imaging have reported similar findings during *imagined* walking by PD subjects, with the left parietal cortex becoming more active than in healthy older adults.^15^ The parietal cortex is associated with sensorimotor integration and gait control, particularly perception of movement^63^ and awareness of an individual’s body in space.^64^ Here, walking with visual cues possibly provides heightened awareness of body position in relation to the space ahead. More specifically, higher theta and alpha power over the parietal cortex in healthy young adults has been associated with greater gait stability during walking.^65,66^ Our results, therefore, may indicate that in PD subjects more cortical control is required for stability when walking with versus without the visual cues.

Higher delta power during walking in PD compared to controls has not been reported in previous event-related EEG walking studies in PD.^67^ However, smaller delta power may represent the stronger attentional processing in parietal cortex required during walking in PD subjects compared to controls.^68^ Similar results have been reported for other clinical cohorts with walking impairments (e.g. following musculoskeletal injury).^69^ Our finding of stronger power in left parietal cortex of PD+FOG subjects, compared to the two other subject groups, may also relate to altered functional brain connectivity reported for PD+FOG subjects in this brain region in resting state functional MRI studies,^70^ possibly impacting bilateral co-ordination of the legs while walking.

### Brain Activity Relationships With Gait

In line with previous studies, in all our participants gait measures improved when walking with visual cues. However, our exploratory correlational analysis showed that brain activity in PD when walking with visual cues was significantly associated with cued gait characteristics, particularly in those with PD+FOG. Specifically, within PD-FOG, better gait with visual cues (e.g. higher foot strike angle) was associated with higher beta power (i.e. a shift to attentional processing) in parietal cortex sources, which may reflect more cognitive control and planned walking with cues.^71,72^ Alternatively in PD+FOG, stronger gamma power (i.e. increased cognitive processing) in occipital lobe sources was associated with faster speed and longer stride length during walking with cues, likely due to increased attentional processing of visual information during cueing as this is a more complex task.^54^ Overall, our results suggest that better gait with visual cues in PD may be related to changes in brain activity, with different PSD responses seen in PD-FOG and PD+FOG that may relate to underlying cognitive processes. This study was the first to explore novel correlational analysis between brain activity, gait and attention, but cautious interpretation should be taken and further robust analysis should be performed in the future.

### Clinical Implications

Addition and use of visual cues is a commonly used and recommended clinical intervention for gait impairment in PD. However, responses to visual cues vary, and little is known about their habitual use. To develop visual cueing interventions that are optimal and targeted for particular gait issues (e.g. FOG), it is useful to understand the underlying brain activity associated with gait response to visual cues. This study has demonstrated that EEG source power, in alpha, beta and gamma bands, of sources within brain regions involved in visual processing is altered during visually cued walking by PD subjects, and these changes are related to cued gait characteristics, particularly in PD+ FOG subjects. These results are compatible with the interpretation that stronger attentional processing is involved in use of visual cues by PD+FOG subjects. Further research is required to understand brain activity in response to introducing various cueing modalities, which could allow for more robust and targeted cueing interventions for individuals. In particular, targeting brain activity and associated attentional deficits in PD subjects with therapeutic interventions such as transcranial direct current stimulation or pharmacological manipulation might lead to gait improvements.

### Study Limitations

There are several limitations to this study that should be noted. Specifically, while 32-channel EEG provides enough data to reduce effects of muscle and other non-brain artefact,^73^ and allows source cluster analysis,^74^ still more scalp channels (e.g. 64 or 128) would allow better source localization of the EEG source signals.^23^ In addition, as ability to stand and walk unassisted was an inclusion criteria in this study, these results may not generalize to later stages of PD. Another limitation, one that affects the majority of the cued walking literature, was that we only examined the immediate effect of cueing on brain activity and gait in PD and did not examine longitudinal or habitual changes that may occur with continued use of cueing. Additionally, this study involved only a limited range of visual function assessments (e.g. visual acuity and contrast sensitivity), measures commonly used within clinical settings. Despite the lack of difference between our groups in visual function, future studies should include a more thorough set of vision assessments (e.g. assessment of depth and motion perception) as other visual functions may have an influence on visual cue response in PD subjects. This was the first study to examine relationships between visually cued brain activity and gait, as well as attention metrics in PD-FOG and PD+FOG, but due to the exploratory nature of our correlational analysis we did not control for multiple comparisons in order to avoid Type II error (false negatives) and this could be performed in future studies with larger cohorts to reduce Type I error risk (false positives).

## Conclusion

This study provides important insights into EEG and walking performance responses to the introduction of visual cues during walking by PD subjects, knowledge that may ultimately help clinicians to enhance safe mobility and reduce falls risk in PD. EEG brain activity within brain regions supporting visual processing is altered in PD when walking with visual cues, particularly in those who self-report FOG. Differences in brain activity in PD subjects during walking may directly relate to their differences in gait performance in response to introduction of visual cues and may relate directly to differences in attentional capabilities of PD+FOG subjects. Future research involving cueing interventions in PD should further examine the brain activity involved in response in order to inform intervention development.
